# The New System of Paying Patients

**Published:** 1921-02-12

**Authors:** 


### GREAT NORTHERN CENTRAL HOSPITAL.

### The New System of Paying Patients.

The Great Northern Hospital serves a large area with a population of over 1,000,000. From experience it is found that the residents may be divided into three categories, each approximately as follows :—

500,000 industrial wage-earners.

350,000 professional or salaried workers, who at times need the services of the hospital in cases of serious illness or operation, and who are able to contribute a moderate amount towards the cost of their maintenance.

150,000 with sufficient means to be able to make such provision for themselves as to be independent of the hospital.

Of the 350,000 professional people probably rather more than half are existing on incomes which have not increased as rapidly as prices in general have advanced, and have therefore little or no margin on which to draw in emergency.

Having arrived at this classification, the hospital has now graded its wards in its main buildings as follows :—

(*a*) General wards, 140 beds.
(*b*) Contributory wards, 62 beds.
(*c*) Private wards, 18 beds.

From these figures it will be seen that the distribution of beds between the 500,000 wage-earners and 350,000 professional people is as follows :—

| | Percentage of Population Needing Hospital Treatment. | Percentage of Beds Available. |
|---|---|---|
| (*a*) 500,000 ... ... | 59% | 64% |
| (*b*) 350,000 ... ... | 41% | 36% |

In the actual working of the scheme there is naturally more elasticity than is suggested by the bare figures, but the net result is probably not very different from that shown by them.

The forms of application for admission provide for a statement by the applicant as to the amount of his income, and taking the income of a single person (without dependants) as a basis, patients are allotted thus to the different categories of wards :—

(*a*) Not exceeding £100 per annum.
(*b*) £100 to £300 per annum.
(*c*) £300 to £500 per annum.

In the general wards necessitous cases are admitted free, but patients who can afford to do so give from 1s. to 20s. per week, according to circumstances. The contributory wards are intended for those unable to meet the full expenses of medical service and nursing at home, and they give while in the hospital from one guinea to four guineas per week, according to their means, as assessed by the Committee. The treatment of patients in the general and contributory wards is undertaken gratuitously by the honorary medical staff.

The private wards, which have just been inaugurated, are intended for the benefit of patients whose means do not allow of the payment of the usual medical and surgical fees, but who are willing to contribute towards the cost of medical services and of maintenance while in the hospital. The payments range from four and a-half to six guineas per week, with a fee for medical service of one guinea per visit—maximum fee for medical service, three guineas per week. These fees are payable to the member of the honorary medical staff actually concerned in the treatment. In the event of an operation a charge of five guineas is payable, which covers the reduced fees of the surgeon and the anæsthetist and the charge for the use of the theatre. By arrangement with a member of the honorary medical staff and the patient concerned, private practitioners attend the hospital to share in the treatment. The patient in this case pays the local medical practitioner's fees. In assessing the amount payable by patients the Committee will arrange for a rebate of 50 per cent. off the medical fees where the income is the minimum for the private wards, or where it exceeds the minimum by not more than £100.

The hospital has also in operation a scale of charges in its out-patient department. Those patients who are able to do so pay 1s. per visit, which entitles them to one week's supply of medicine and dressings; 6d. is payable for each additional week's requirements; 5s. is charged for minor operations, 3s. per attendance for *x*-ray treatment or massage, and 2s. 6d. for an anæsthetic. It should be added that the almoner is empowered to remit any of these charges or to allow a rebate in any necessitous case.

The Great Northern has had the courage to take a very real step forward, and the progress of their scheme will be closely followed by all who are interested in our present hospital system. We wish their efforts every success.

